# Effects of Zr Addition on Strengthening Mechanisms of Al-Alloyed High-Cr ODS Steels

**DOI:** 10.3390/ma11010118

**Published:** 2018-01-12

**Authors:** Jian Ren, Liming Yu, Yongchang Liu, Chenxi Liu, Huijun Li, Jiefeng Wu

**Affiliations:** 1 State Key Lab of Hydraulic Engineering Simulation and Safety, Tianjin Key Lab of Composite and Functional Materials, Tianjin University, Tianjin 300072, China; zzusimon@163.com (J.R.); ycliu@tju.edu.cn (Y.L.); cxliutju@163.com (C.L.); huijun@uow.edu.au (H.L.); 2 Institute of Plasma Physics, Chinese Academy of Sciences, Hefei 230031, China; jfw@ipp.ac.cn

**Keywords:** Zr addition, ODS, tensile properties, strengthening mechanism

## Abstract

Oxide dispersion strengthened (ODS) steels with different contents of zirconium (denoted as 16Cr ODS, 16Cr-0.3Zr ODS and 16Cr-0.6Zr ODS) were fabricated to investigate the effects of Zr on strengthening mechanism of Al-alloyed 16Cr ODS steel. Electron backscatter diffraction (EBSD) results show that the mean grain size of ODS steels could be decreased by Zr addition. Transmission electron microscope (TEM) results indicate that Zr addition could increase the number density but decrease the mean diameter and inter-particle spacing of oxide particles. Furthermore, it is also found that in addition to Y-Al-O nanoparticles, Y-Zr-O oxides with finer size were observed in 16Cr-0.3Zr ODS and 16Cr-0.6Zr ODS steels. These changes in microstructure significantly increase the yield strength (YS) and ultimate tensile strength (UTS) of ODS steels through mechanisms of grain boundary strengthening and dispersion strengthening.

## 1. Introduction

Oxide dispersion strengthened (ODS) steels have been considered as promising fuel cladding materials for advanced nuclear systems and blanket materials for fusion power systems [1]. Due to hard working conditions at high temperature corrosion and radiation environment, the ODS steels should exhibit excellent mechanical properties combining with good corrosion and irradiation resistance [2,3]. 

The microstructure and mechanical properties of ODS steels depend not only on the manufacturing process but also on their proper chemical composition. ODS steels with ferritic-martensitic and ferritic matrix were designed in previous studies [4,5,6,7,8]. In view of favorable mechanical properties and good corrosion resistance, high Cr (15~20 wt %) strategy is usually adopted in composition design. High-Cr ferritic ODS steels, such as commercial ODS PM2000 (20 wt % Cr and 5.5 wt % Al) and MA956 (20 wt % Cr and 4.5 wt % Al), usually contain a certain amount of Al [8]. The aluminum can improve the corrosion and oxidation resistance of ODS steels by forming dense alumina layer [9,10]. Meanwhile, the aluminum is also used as one of the alloying element that decreases the tensile strain anisotropy and eliminates embrittlement at 475 °C of Fe-Cr alloys [11,12,13,14].

However, the aluminum addition enables easy formation of large Y-Al-O precipitation particles, deteriorating the mechanical properties of ODS steels [15,16,17,18,19,20]. Zhang et al. found that the 14Cr-Ti ODS steel exhibited better tensile strength than 14Cr-Al ODS steel. This might be related to the formation of dense Y_2_Ti_2_O_7_ particles in 14Cr-Ti ODS steel, while larger Y-Al-O particles with lower number density, such as yttrium-aluminum hexagonal YAlO_3_ (YAH) and yttrium-aluminum monoclinic Y_4_Al_2_O_9_ (YAM) were formed in 14Cr-Al ODS steel [20]. Dong et al. investigated the effects of hafnium (Hf) addition on the microstructure and mechanical performance of Al-alloyed high-Cr ODS steels, and found that a large number of refined Y-Hf-O precipitates were formed instead of coarse Y-Al-O oxide particles, which apparently increased the tensile strength [15]. Isselin et al. studied the influence of Zr addition on microstructure of ODS steels, and found that Zr addition strongly suppressed the formation of Al and Y oxides in micro scale [21]. In general, Ti, Hf and Zr all have positive effects on optimizing the microstructure of high-Cr ODS steels. The first principle calculation results indicate that the binding energies of Y-Zr-O and Y-Hf-O clusters are higher than those of Y-Al-O and Y-Ti-O clusters in ferrite matrix [22,23]. Therefore, it seems that Zr and Hf are more effective than Ti. The zirconium has prominent nuclear properties and smaller thermal neutron capture cross-section than Hf [24]. From this point of view, Zr is a more appropriate choice to improve the mechanical properties of Al-alloyed high-Cr ODS steels. It is necessary to further investigative the effect of Zr addition on microstructure and tensile strengths of Al-alloyed high-Cr ODS steels.

In this paper, effects of Zr addition on strengthening mechanisms of Al-alloyed 16Cr ODS steel were investigated. Three compositions of ODS steels (16Cr ODS, 16Cr-0.3Zr ODS and 16Cr-0.6Zr ODS) were designed and fabricated through mechanical alloying (MA) and hot isostatic pressing (HIP). Refinements of precipitates and grains with Zr addition were discussed in detail, as well as tensile properties. Furthermore, improvement of YS and UTS of two ODS steels with Zr addition was explained based on different strengthening mechanisms.

## 2. Experimental

### 2.1. Material

The argon-gas atomized pre-alloyed Fe-16Cr-3Al-1.5W powders (average size of 50 μm) were mixed with 0.35 wt % Y_2_O_3_ (average size of 40 nm) and different amounts of Zr (0 wt %, 0.3 wt % and 0.6 wt %), respectively. The Zr powders have a purity of 99.9% and the mean particle size is about 10 μm. The mixed powders were mechanically milled on a high-energy planetary ball mill (QM-2SP12, Nanjing NanDa Instrument Plant, Nanjing, China) at a rotating speed of 250 rpm for 30 h, with high purity of Ar as protection. The weight ratio of ball to powder was 10:1. The as-milled powders were sealed in mild-steel cans and degassed at 450 °C until the vacuum degree of 0.002 Pa. Then, the powders were consolidated by HIP at 1150 °C for 3 h under the pressure of 150 MPa. The carbon and nitrogen are strictly controlled in the fabrication process. Table 1 shows the compositions design of three ODS steels investigated in this study.

### 2.2. Microstructure Characterization

The matrix microstructure and oxide particles of ODS steels were examined by a FEI Quanta 650F scanning electron microscope (SEM, FEI, Hillsboro, OR, USA) equipped with electron backscattered diffraction (EBSD, FEI, Hillsboro, OR, USA) (HKL Channel 5) and a transmission electron microscope (TEM, JEM-2100F, JEOL, Tokyo, Japan) equipped with an energy-dispersive spectroscope (EDS). The identification of the oxides was performed by high-resolution transmission electron microscope (HRTEM). The EBSD samples with 10 mm in length and 1 mm in thickness, were prepared by electrolytic polishing. TEM samples with 3 mm in diameter, were punched in 50 μm thickness slice, and then thinned by mechanical grinding. Subsequently, the TEM discs were etched by a twin-jet electro-polishing machine with solution of 5% perchloric acid and 95% ethanol at −20 °C. The dislocation density of three ODS steels was evaluated by observing TEM micrographs through the method proposed by Pešička [25].

### 2.3. Tensile Test

Tensile test was carried out on a 300 kN electronic tensile testing machine (GNT300, NCS, Shanghai, China) at a nominal strain rate of 7 × 10^−4^ s^−1^ at room temperature (RT). The rod shaped tensile samples with a gauge length of 25 mm and a diameter of 8 mm were prepared from the as-HIPed steels bars.

## 3. Results and Discussion

### 3.1. Microstructure/Substructure Examination

#### 3.1.1. Grain Morphologies

**Figure 1:**
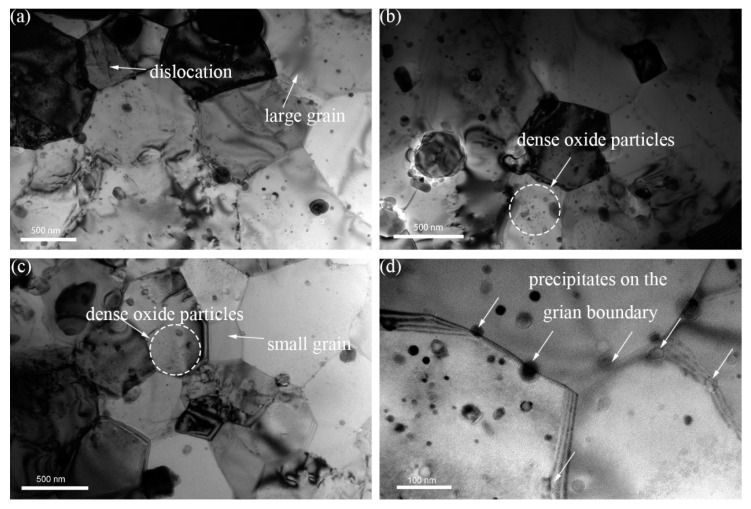
TEM images of microstructure/substructure in: (**a**) 16Cr ODS; (**b**) 16Cr-0.3Zr ODS; and (**c**) 16Cr-0.6Zr ODS steels; and (**d**) precipitates on the grain boundary of 16Cr-0.6Zr ODS steel.

Figure 1a–c shows the microstructure/substructure of 16Cr ODS, 16Cr-0.3Zr ODS and 16Cr-0.6Zr ODS steels, respectively. The matrix microstructure all consists of equiaxed ferrite grains with different sizes. Both large and small precipitates can be observed in the TEM image. Furthermore, a high density of oxide particles within the grain can be observed in the small grains of 16Cr-0.3Zr ODS and 16Cr-0.6Zr ODS steels. In Figure 1d, some precipitates with a diameter of ~10 nm are located at the grain boundary of 16Cr-0.6Zr ODS steel, which is also observed in the other two ODS steels. This pinning effect might be related to the grain boundary migration during HIP.

**Figure 2:**
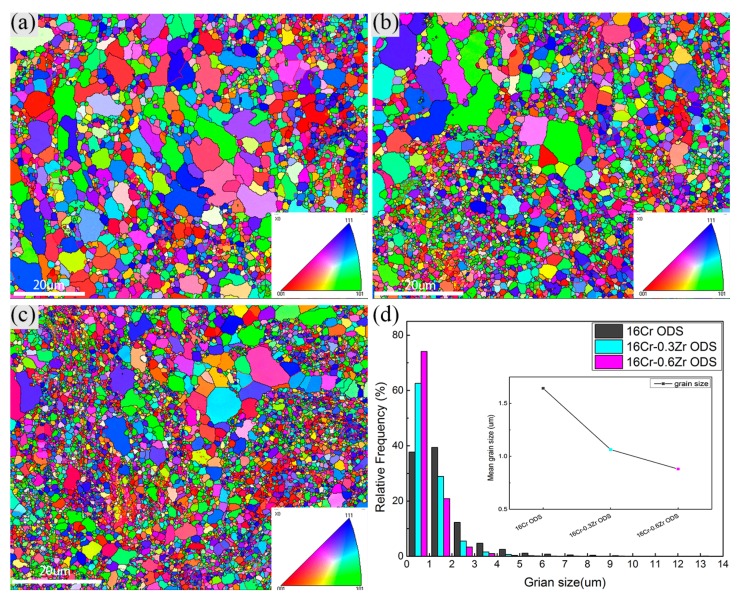
Inverse pole figure (IPF) and grain boundary maps of: (**a**) 16Cr ODS; (**b**) 16Cr-0.3Zr ODS; and (**c**) 16Cr-0.6Zr ODS steels; and (**d**) statistical results of grain size distribution in these three ODS steels.

**Figure 3:**
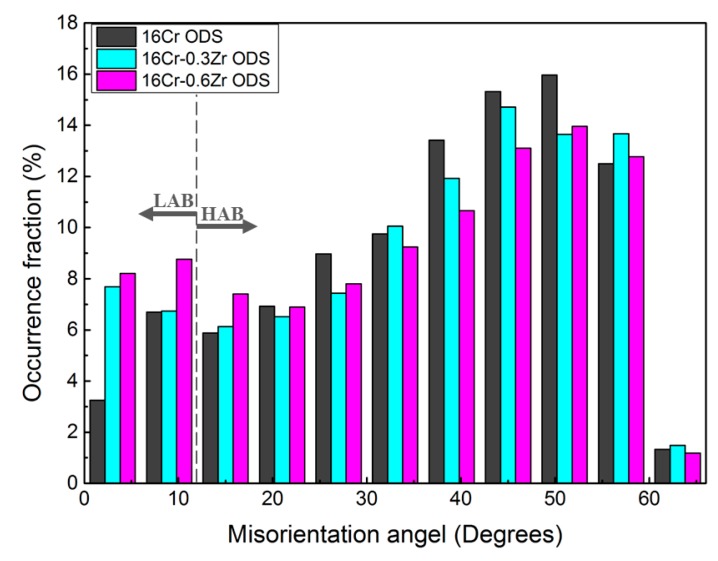
Grain misorientation distribution of three ODS steels.

Figure 2a–c shows the inverse pole figure (IPF) and grain boundary maps of these ODS steels by EBSD. The grains in the ODS steels all exhibit the random orientation. The co-existence of large and small grains in the map is related to the abonormal grain growth during consolidation. Figure 2d shows the size distribution of grains in the three ODS steels. The mean grain sizes are determined as 1.64 ± 1.37 µm, 1.06 ± 0.84 µm and 0.88 ± 0.64 µm for 16Cr ODS, 16Cr-0.3Zr ODS and 16Cr-0.6Zr ODS steel, respectively. The standard deviation is relatively large because of the existence of some large sized grains in three ODS steels. It can be concluded that Zr addition could significantly decrease the grain size. Figure 3 shows the grain misorientation distribution of three ODS steels. All three ODS steels exhibit a uniform grain misorientation distribution. However, the occurrence fraction of low angle grain boundaries (LAB) are determined as 10.0%, 14.4% and 17% for 16Cr ODS, 16Cr-0.3Zr ODS and 16Cr-0.6Zr ODS steels, respectively. This result indicates that Zr addition could increase the LAB of Al-alloyed ODS steels.

#### 3.1.2. Dislocation Density

**Figure 4:**
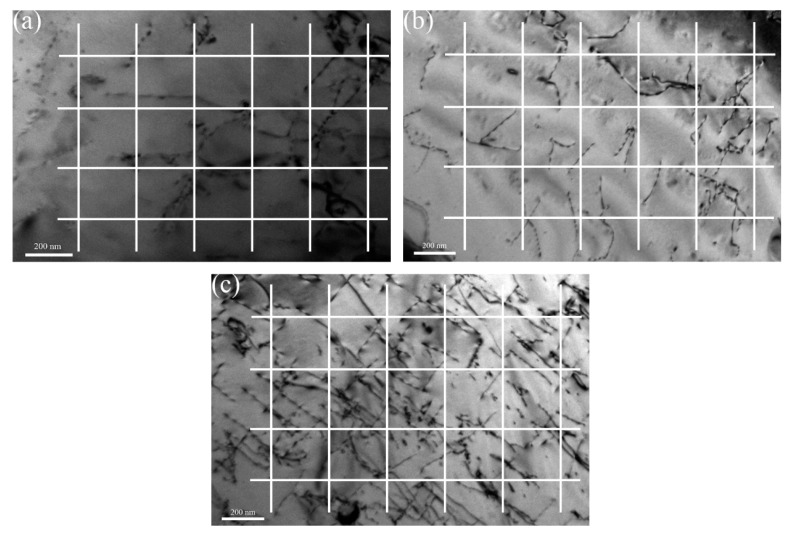
Sketch of the method for the measurement of dislocation density in: (**a**) 16Cr ODS steel; (**b**) 16Cr-0.3Zr ODS steel; and (**c**) 16Cr-0.6Zr ODS steel.

The interaction between precipitates and dislocation plays an important role in strengthening ODS steels. The measurement of dislocation density is based on the method proposed by Pešička [25]. Figure 4a shows the distribution of dislocation in 16Cr ODS steel. A grid consisting of four horizontal and five vertical lines are superimposed on the TEM micrographs. The numbers $n_{h}$ and $n_{v}$ of intersections of dislocations with the horizontal and vertical grid lines are counted. TEM foil thickness (t) of the observed dislocation is usually between 180 and 220 nm (dislocations were not easily found in thinner foils, which were often bent and associated with high internal stresses, neither could dislocation densities be determined from thicker foils, where only dark micrograph with low contrast were obtained). With the total lengths of the horizontal ($\sum L_{h}$) and vertical test lines ($\sum L_{v}$) from all micrographs taken for one materials state the dislocation density ($\rho_{Disloc}$), the $\rho_{Disloc}$ could be obtained as [25,26]:

(1) $$\rho_{Disloc}=\frac{1}{t}\left(\frac{\sum n_{v}}{\sum L_{v}}+\frac{\sum n_{h}}{\sum L_{h}}\right)$$

The resulting $\rho_{Disloc}$ is $5.0\times 10^{13}$ for 16Cr ODS steel. The same methods were used to identify the dislocation density in 16Cr-0.3Zr and 16Cr-0.6Zr ODS steels, as shown in Figure 4b,c. The calculated results are $5.6\times 10^{13}$ and $5.8\times 10^{13}$ for 16Cr-0.3Zr ODS and 16Cr-0.6Zr ODS steels, respectively. From the results, Zr addition slightly increases the dislocation density of ODS steels.

#### 3.1.3. Spatial and Size Distributions of Oxide Particles

**Figure 5:**
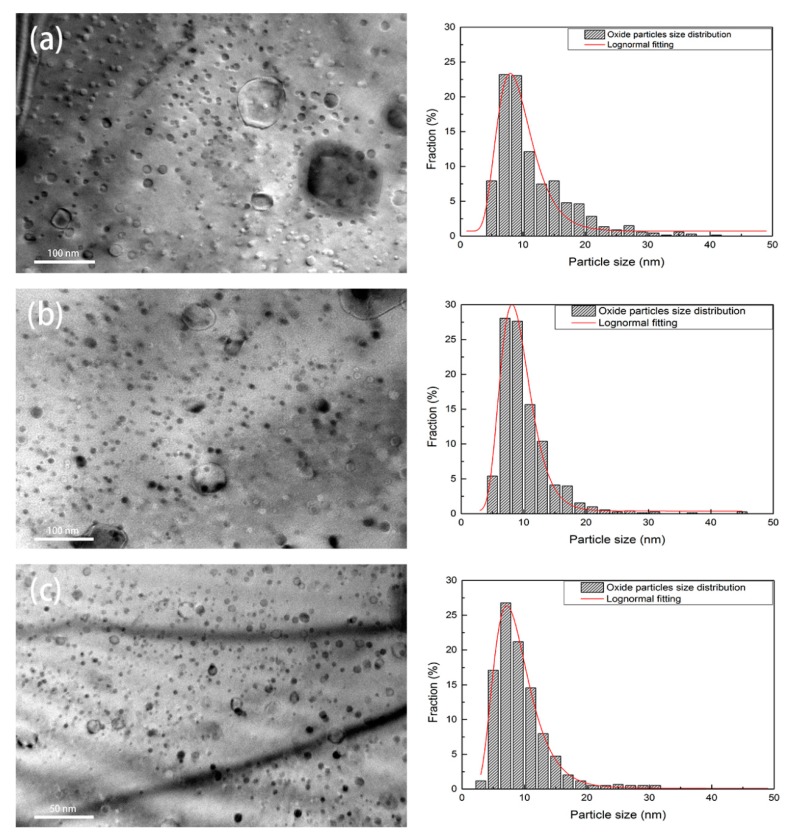
TEM images and size distribution of the oxide nanoparticles in: (**a**) 16Cr ODS steel; (**b**) 16Cr-0.3Zr ODS steel; and (**c**) 16Cr-0.6Zr ODS steel.

Figure 5 shows the typical morphologies and distribution of oxide nanoparticles in three ODS steels. Oxide nanoparticles with a high number density distribute homogeneously within the grains. The statistical results indicate that the oxide nanoparticles with diameters from 2 nm to 10 nm account for 58% in 16Cr-0.3Zr ODS and 70% in 16Cr-0.6Zr ODS steels, compared to less than 50% in 16Cr ODS steel. Based on the calculation results of more than 1000 oxide nanoparticles in each TEM observation, the mean diameter ($d_{p}$), mean inter-particle spacing (λ) and number density ($n_{V}$) of oxides in three ODS steels can be obtained, as illustrated in Table 2. It can be concluded that Zr addition can increase the $n_{V}$, while decreasing $d_{p}$ and λ of oxide nanoparticles in ODS steels. The oxide nanoparticles in the matrix of ODS steels can hinder the grain boundary migration and dislocations movement by pinning effect, thus the higher number density and finer size of the oxide particles is related to the refinement of grain size (see Figure 2) and increase of LAB (see Figure 3) in two Zr-contained ODS steels [27].

#### 3.1.4. Crystal Structures of Oxide Particles Studied by HRTEM

**Figure 6:**
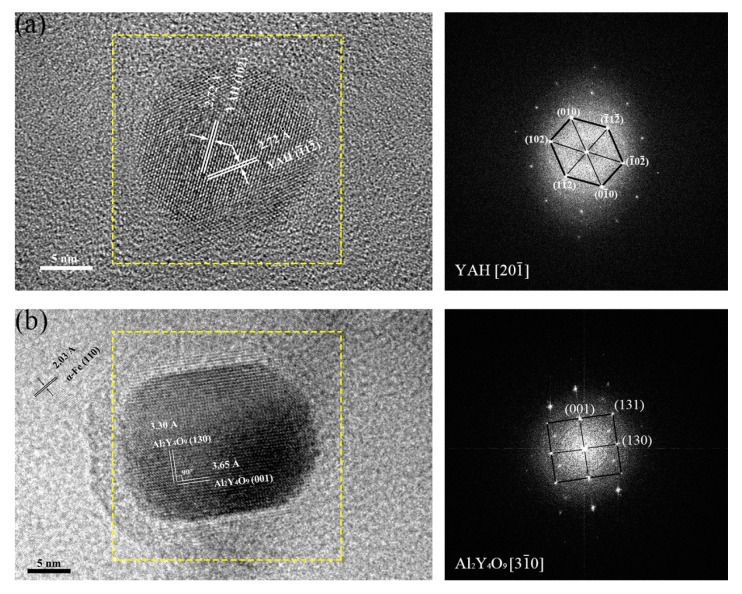
HRTEM micrographs and corresponding FFT images of oxide nanoparticles in 16Cr ODS steel.

In this study, high resolution transmission electron microscope (HRTEM) technique and fast Fourier transformation (FFT) method was used to identify the types of oxides in these ODS steels. Figure 6 displays the HRTEM and FFT images of two oxide nanoparticles in 16Cr ODS steel. The oxide particles were identified as YAH (see Figure 6a) and YAM (see Figure 6b), which correspond to the results found in other studies [16,17].

**Figure 7:**
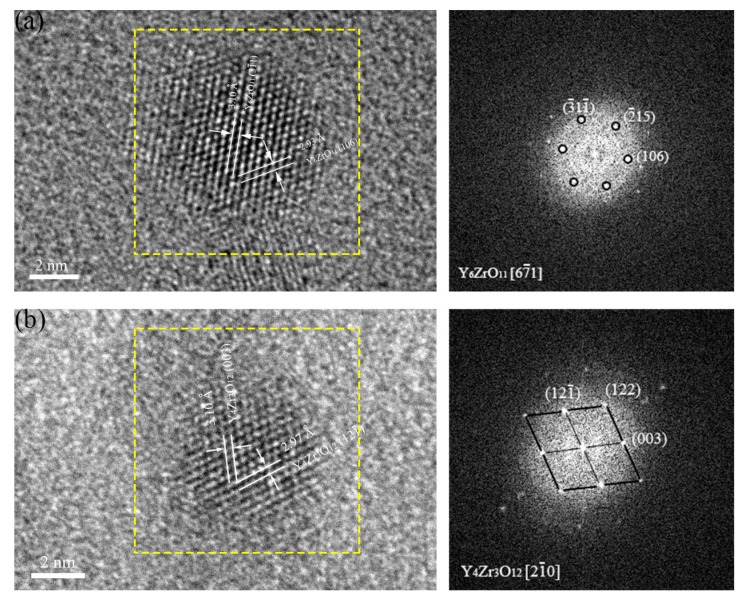
HRTEM micrographs and corresponding FFT images of oxide nanoparticles in: (**a**) 16Cr-0.3Zr ODS steel; and (**b**) 16Cr-0.6Zr ODS steel.

**Figure 8:**
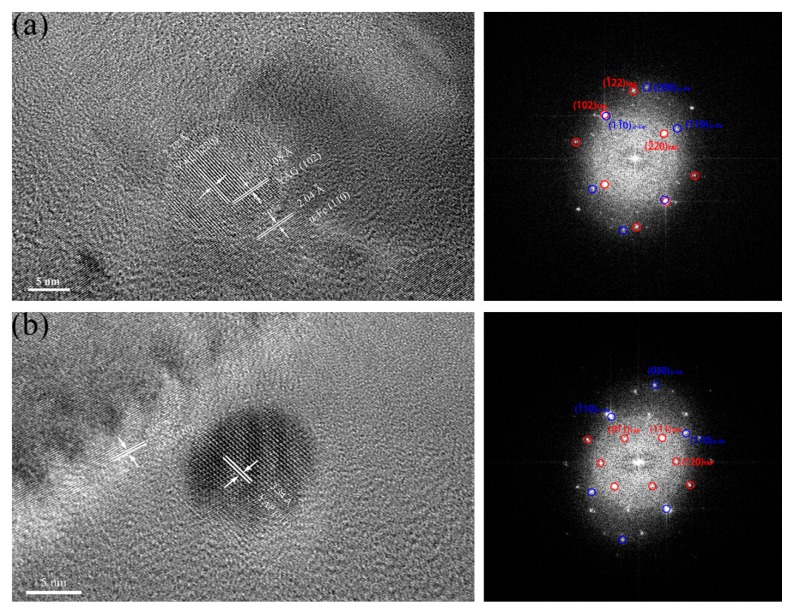
HRTEM micrographs and corresponding FFT images of Y-Al-O oxides in: (**a**) 16Cr-0.3Zr ODS steel; and (**b**) 16Cr-0.6Zr ODS steel.

In Figure 7a, the oxide particle with a diameter of 5 nm is identified to be Y_6_ZrO_11_ in 16Cr-0.3Zr ODS steel. The measured interplanar distances of the oxide (2.93 and 3.10 Å) are consistent with those of $\left(3\bar{1\;}1\right)$ and (106) planes of Y_6_ZrO_11_ (JCPDS: 36-0196, hexagonal structure). Another oxide particle with a diameter of 4.5 nm is identified to be Y_4_Zr_3_O_12_ in Figure 6b. The measured interplanar distances of the oxide (2.97 and 3.10 Å) are consistent with those of $\left(12\bar{1\;}\right)$ and (003) planes of Y_4_Zr_3_O_12_ (JCPDS: 32-1500, hexagonal structure). In addition to Y-Zr-O nanoparticles, Y-Al-O oxides with large sizes are also observed in two Zr-contained ODS steels, as presented in Figure 8. The nanoparticle with a diameter of 15 nm is identified as yttrium–aluminum–garnet (YAG, Y_3_Al_5_O_12_) in Figure 8a and the nanoparticle with a diameter of 12 nm is identified as yttrium–aluminum–perovskite (YAP, YAlO_3_) in Figure 8b. It is found that the oxides with smaller size tend to be Y-Zr-O particles while the mostly large sized oxides are Y-Al-O particles. It has been illustrated that the binding energy of Y-Zr-O clusters are higher than Y-Al-O clusters, thus the formation of Y-Zr-O would be more stable than Y-Al-O. Therefore, the coarsening of Y-Zr-O clusters during process of HIP is not easy.

### 3.2. Mechanical Properties

**Figure 9:**
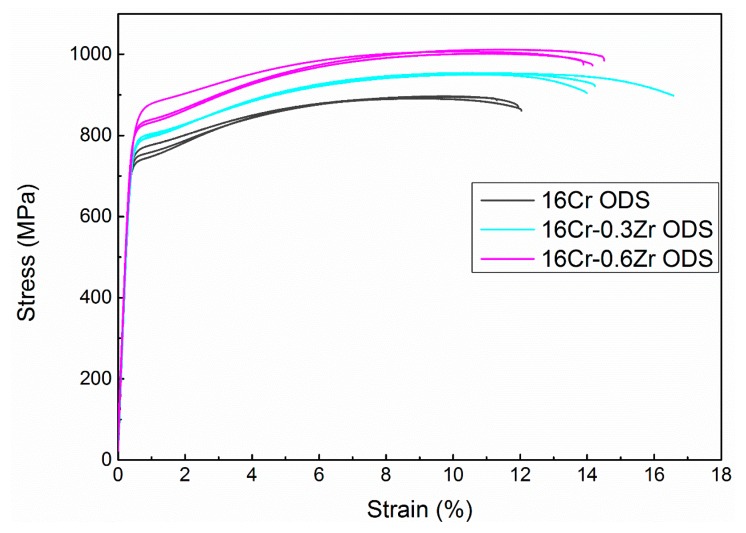
Strain–stress curves of 16Cr ODS, 16Cr-0.3Zr and 16Cr-0.6Zr ODS steels at room temperature.

Figure 9 presents the room temperature tensile strain–stress curves of three ODS steels. Three tensile samples were carried out for each ODS steel. The yield strength (YS) and ultimate tensile strength (UTS) of 16Cr ODS are determined as 743 ± 4 and 894 ± 3 MPa, respectively. The YS and UTS of 16Cr-0.3Zr ODS increased to 773 ± 3 and 952 ± 2 MPa, respectively. The YS and UTS increased to 834 ± 6 and 1007 ± 5 MPa for 16Cr-0.6Zr ODS, respectively. The EBSD results indicate that the grain size of two ODS samples are smaller than 16Cr ODS sample. In fact, grain size has a significant effect on the ductility of ODS steels. The ODS steels would present better ductility after refinement of the grain size. This phenomenon might be explained as follows. Firstly, the grain boundary area is increased after refinement of grain size. The interface bonding strength would also be enhanced. Secondly, more interface would act as wall to hinder the movement of microcracks in the matrix. Finally, fine grain structure contributes to the well-distributed microstructure. These effects might be beneficial to the plastic deformation of ODS steels. Therefore, refinement of grain size would increase the ductility of the two Zr-contained ODS steels. The enhancement in UTS and YS with Zr addition is related to the refinement of precipitates and decrease in grain size. 

To evaluate the contributions of microstructure parameters to the YS of three ODS steels and further compare the detail strengthening mechanisms with Zr addition, the following equation is used [28,29]:

(2) $$\sigma_{y}=\sigma_{0}+\sigma_{ss}+\sigma_{GB}+\sqrt{\sigma_{Disloc}^{2}+\sigma_{Particles}^{2}}$$

where $\sigma_{y}$ is the yield strength, with $\sigma_{0}$ the Peierls–Nabarro force, $\sigma_{SS}$ the solid solution contributions, $\sigma_{GB}$ the contribution of grain boundaries, $\sigma_{Disloc}$ the dislocation forest hardening and $\sigma_{Particles}$ the contribution of nanoparticles.

The Peierls–Nabarro stress (lattice resistance) is commonly expressed by the following equation [29]:

(3) $$\sigma_{0}=\frac{2M\mu }{1-\nu }\exp\left(\frac{-2\pi a}{b(1-\nu )}\right)$$

where μ and ν are the shear modulus and the Poisson coefficient, and are 84 GPa and 0.366, respectively. Taylor factor M, Burgers vector b, and the lattice parameter of pure iron a are determined as 3, 0.248 nm and 0.287 nm, respectively [30,31]. Therefore, the calculated result of $\sigma_{0}$ is 8.31 MPa.

The solid solution strengthening term includes both the interstitial strengthening from carbon and nitrogen and the substitution strengthening from substitutional elements in the matrix. In this study, the carbon and nitrogen are strictly controlled during the processes of MA and HIP, therefore a limited interstitial strengthening would be estimated. Here, we simply consider the contribution of substitutional strengthening from substitutional element Cr, W and Al. The strength increment $\sigma_{SS}$ due to the substitutional alloying elements in bcc iron can be expressed as [32]:

(4) $$\sigma_{ss}=0.00689kX^{n}$$

where $\sigma_{SS}$ is strength in megapascal (MPa), X is equilibrium concentration of substitutional elements in atomic percent, *n* is 0.75 for all the elements investigated, value 0.00689 is for converting psi (pound per square inch) to MPa and k is the strengthening coefficient. The constant k of Cr, W and Al are 1400, 11,000 and 4000, respectively [32]. The calculated $\sigma_{SS}$ is determined as 225.79 MPa for three ODS steels.

The grain boundaries strengthening $\sigma_{GB}$ can be expressed as Hall–Petch model, which is generally inversely proportional to the square root of the mean grain size [29]:

(5) $$\sigma_{GB}=\frac{k_{GB}}{\sqrt{D}}$$

where $k_{GB}$ is a microstructural parameter (0.307 $MPa\cdot \sqrt{m}$) and D the mean grain size. The mean grain sizes are determined as 1.64 µm, 1.06 µm and 0.88 µm for 16Cr ODS, 16Cr-0.3Zr ODS and 16Cr-0.6Zr ODS steels in Section 3.1.1 by EBSD, respectively. The grain boundaries strengthening $\sigma_{GB}$ are calculated as 239.73 MPa, 298.18 MPa and 327.26 MPa for 16Cr ODS, 16Cr-0.3Zr ODS and 16Cr-0.6Zr ODS, respectively.

The dislocation forest hardening $\sigma_{Disloc}$ can be described as [33]:

(6) $$\sigma_{Disloc}=M\alpha \mu b\sqrt{\rho_{Disloc}}$$

where α is a constant (0.33) [34]. M, μ and b are the same parameters used in the calculation of $\sigma_{0}$. The $\rho_{Disloc}$ is the dislocation density measured in Section 3.1.2. The dislocation forest hardening $\sigma_{Disloc}$ are calculated as 145.83, 154.33 and 157.07 MPa for 16Cr ODS, 16Cr-0.3Zr ODS and 16Cr-0.6Zr ODS, respectively.

Finally, the contribution of oxide nanoparticles was estimated by [35]:

(7) $$\sigma_{Particles}=\frac{0.81M\mu b}{2\pi {\left(1-\nu \right)}^{1/2}}\frac{\ln\left(2\sqrt{\frac{2}{3}}r/2b\right)}{\left(\sqrt{\frac{2\pi }{3f}}\cdot r\right)}$$

where r and f are the mean radius and volume fraction of oxide nanoparticles. M, μ, ν and b are the same parameters used in the calculation of $\sigma_{0}$. The mean diameters and inter-particle spacing of oxide particles $d_{p}$ and λ of three ODS steels are listed in Table 2, while the mean radius r can be reached as $r=d_{p}/2$. The volume fraction of oxide nanoparticles f can be expressed by $f={\left(2r/\lambda \right)}^{3}$ [36]. The calculated results of $\sigma_{Particles}$ are 201.46, 244.43 and 262.30 MPa for 16Cr ODS, 16Cr-0.3Zr ODS and 16Cr-0.6Zr ODS, respectively.

**Figure 10:**
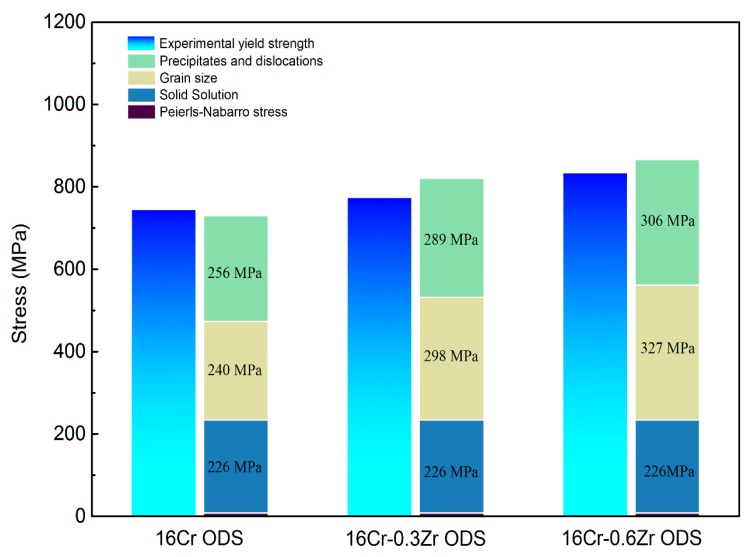
Comparison between the experimental and the estimated yield strength represented by components from Peierls-Nabarro stress, solid solution strengthening, grain boundaries strengthening, dislocation forest hardening and dispersion strengthening.

After evaluation of each of the above specific strengthening contributions, a comparison between the experimental yield strength and the estimated one represented by components from Peierls–Nabarro stress, solid solution strengthening, grain boundaries strengthening, dislocation forest hardening and dispersion strengthening is shown in Figure 10. The insert number in the histogram represent the detail numbers of each strengthening mechanism contributed to the calculated YS. In all three ODS steels, the major strengthening mechanisms are grain boundary strengthening, dispersion strengthening and dislocation forest hardening. The dispersion strengthening and dislocation hardening make the major contribution to the YS of 16Cr ODS steel. However, with decrease in grain size, the grain boundary strengthening plays the major role in 16Cr-0.3Zr ODS and 16Cr-0.6Zr ODS steels. The increase in YS with Zr addition is related to the grain boundary strengthening and dispersion strengthening mechanisms. Decrease in grain size contributes to the grain boundary strengthening ($\sigma_{GB}$) and the refinement in oxide particle is beneficial to the dispersion strengthening ($\sigma_{Particles}$). The improvement of YS through refinement of oxide particles and reduction in grain size by Zr addition is evident. 

## 4. Conclusions

Three ODS steels with or without Zr addition were successfully fabricated by mechanical alloying and subsequent HIP. The effect of Zr addition on strengthening mechanisms of Al-alloyed 16Cr ODS steel have been investigated in this work. Some conclusions can be summarized as follows:

(1) The oxides in 16Cr ODS steel are mainly Y-Al-O nanoparticles; however, with Zr addition in 16Cr-0.3Zr ODS and 16Cr-0.6Zr ODS steels, some precipitates are identified as Y-Zr-O nanoparticles. Y-Zr-O nanoparticles exhibit a smaller size compared to Y-Al-O particles.
(2) Smaller Y-Zr-O oxide nanoparticles in Zr-contained ODS steels can lead to the formation of more homogenous and dispersive oxides. The oxides in Zr-contained ODS steels exhibit a smaller size, higher number density and more homogeneous distribution compared to those in 16Cr ODS. These refined oxides inhabit the grain growth during HIP and lead to a more refined grain size. Therefore, the Zr addition could decrease the grain size and increase the LAB of Al-alloyed 16Cr ODS steels.
(3) Decrease in grain size and refinement of oxide particle increase the yield strength of ODS steels with Zr addition. The major strengthening mechanisms change from dispersion strengthening and dislocation hardening to grain boundary strengthening in 16Cr-0.3Zr and 16Cr-0.6Zr ODS steels when compared to 16Cr ODS steel.

## Figures and Tables

**Table 1:** Nominal composition (wt %) of the three ODS steels.

ODS Samples	Fe	Cr	Al	W	Zr	Y_2_O_3_
16Cr-ODS	Bal.	16	3	1.5	-	0.35
16Cr-0.3Zr-ODS	Bal.	16	3	1.5	0.3	0.35
16Cr-0.6Zr-ODS	Bal.	16	3	1.5	0.6	0.35

**Table 2:** The average diameter $d_{p}$, inter-particle spacing λ and number density $n_{V}$ of oxide particles in 16Cr ODS steel, 16Cr-0.3Zr ODS steel and 16Cr-0.6Zr ODS steel.

ODS Samples	$d_{p}$ (nm)	λ (nm)	$n_{V}$ (m^−3^)
16Cr ODS	11.59 ± 0.60	30.03 ± 1.50	1.33 × 20^23^ ± 0.5 × 20^23^
16Cr-0.3Zr ODS	10.21 ± 0.45	25.56 ± 1.75	1.81 × 20^23^ ± 0.8 × 20^23^
16Cr-0.6Zr ODS	9.5 ± 0.50	23.33 ± 2.0	2.04 × 20^23^ ± 0.6 × 20^23^
